# Preclinical electrogastrography in experimental pigs

**DOI:** 10.2478/v10102-010-0011-5

**Published:** 2010-06

**Authors:** Jaroslav Květina, Jithinraj Edakkanambeth Varayil, Shahzad Marghoob Ali, Martin Kuneš, Jan Bureš, Ilja Tachecí, Stanislav Rejchrt, Marcela Kopáčová

**Affiliations:** 1 Institute of Experimental Biopharmaceutics, Joint Research Centre of Czech Academy of Sciences and PRO.MED.CS Praha as, Hradec Králové, Czech Republic; 2 University Department of Gastroenterology, Charles University in Praha, Faculty of Medicine at Hradec Králové, Hradec Králové, Czech Republic; 3 2^nd^ Department of Internal Medicine, Charles University in Praha, Faculty of Medicine, University Teaching Hospital, Hradec Králové, Czech Republic

**Keywords:** electrogastrography, preclinical studies, experimental pig

## Abstract

Surface electrogastrography (EGG) is a non-invasive means of recording gastric myoelectric activity or slow waves from cutaneous leads placed over the stomach. This paper provides a comprehensive review of preclinical EGG. Our group recently set up and worked out the methods for EGG in experimental pigs. We gained our initial experience in the use of EGG in assessment of porcine gastric myoelectric activity after volume challenge and after intragastric administration of itopride and erythromycin. The mean dominant frequency in pigs is comparable with that found in humans. EGG in experimental pigs is feasible. Experimental EGG is an important basis for further preclinical projects in pharmacology and toxicology.

## Introduction

Surface electrogastrography (EGG) is a non-invasive means of recording gastric myoelectrical activity or slow waves from cutaneous leads placed over the stomach. Neuromuscular activities of the stomach generate electrical phenomena termed “gastric slow waves”. The gastric myoelectrical activity is made up of two types of electrical signals termed slow waves or electrical control activity and superimposed spikes also called electrical response activity. The gastric pacemaker is located at the greater curvature of stomach adjacent to the junction between the fundus and the body (Chen & McCallum, 1994; Parkman *et al*., 2003; Chang, 2005).

## Assessment of motor function of the stomach

Normal gastrointestinal motor function is a complex series of events that requires coordination of the sympathetic and parasympathetic nervous systems, neurons within the stomach and intestine, as well as the smooth muscle cells of the gut. Several tools are available to evaluate motor function and related disorders, like EGG, gastric emptying tests (gastric scintigraphy, breath tests using ^13^C-octanoic acid, ^13^C-acetate or spirulina, a plant based protein), gastroduodenal manometry, electronic barostat and planimetry of the gastric antrum by abdominal ultrasonography or magnetic resonance. Simultaneous measurement of intra-luminal pH and pressure by a special capsule is a new method of investigation (Camileri *et al*., 1998, 2010). The capsule, when swallowed, can simultaneously measure phasic pressure amplitudes and pH as it traverses different segments of the gastrointestinal tract. The characteristic change in pH between the stomach and the small intestine provides an indication of the gastric emptying time for a non-digestible solid >1cm long (Camilleri, 2010).

Fasted and postprandial recording running spectrum percent activity and changes in the aplitude (power analysis) are the major measures for the evaluation of EGG. According to the American Motility Society (Parkman *et al*., 2003), normal EGG frequency (2.0–4.0 cycles per minute) should comprise ≥70% of recording time in humans. The possible relationship between EGG and gastric emptying also remains controversial (Mintchev *et al*., 1993; Bortolotti, 1998; Sanmiguel *et al*., 1998; Sha *et al*., 2009). The major parameters of EGG and gastric emptying measures (*e.g.* half-life of elimination in ^13^C-octanoic acid breath test) mutually correlate in healthy humans (Bureš *et al*., 2007). There could probably be a difference between healthy status and disease (*i.e.* diabetes mellitus, systemic sclerosis, functional dyspepsia, eating disorders, gastrectomy etc.) (Chen & McCallum, 1994; Diamanti *et al*., 2003; Bureš *et al*., 2007, 2008; Camileri *et al*., 2010).

In humans, pathological changes at EGG, *i.e.* alterations in frequency (bradygastria, tachygastria, or mixed, dysrhytmias) and reduction in the amplitude of the postprandial electrical signal are seen in patients with idiopathic and diabetic gastroparesis, functional dyspepsia, anorexia nervosa, nausea of pregnancy, vector/motor sickness, Helicobacter pylori infection, status after gastric surgery. Dysrhythmias have also been described in patients with functional dyspepsia, with or without evidence of gastric stasis (Chen & McCallum, 1994, 2006; Bureš *et al*., 1998, 2008; Sanmiguel *et al*., 1998; Ogawa *et al*., 2004). In healthy people, dominant frequency varies during the day (maximum frequency at midday and minimum frequency during the night) (Lindberg *et al*., 1996). Age, sex (including menstrual cycle in women) and body-mass index might influence EGG in humans (Real Martínez *et al*., 2001; Tojl *et al*., 2007). However, even more physiological and/or social events can influence EGG in humans, like meals and volume challenge, ethanol intake, listening to enjoyable music etc. (Levanon *et al*., 1998; Lin *et al*., 2007; Kobak & Bor, 2007).

## Importance of preclinical studies

In humans, several drugs were tested to influence myoelectrical activity of the stomach, like prokinetics (cisapride, tegaserod, domperidon), muscarinic M3 receptor agonists (cevimeline), hyoscine butylbromide, fentanyl and others (Chiba *et al*., 2007; Walldén *et al*., 2008; Americo *et al*., 2009). Interestingly, probiotics and prebiotics might influence EGG in humans too. Probiotic *Lactobacillus reuteri* stimulated gastric emptying and improved maturation of the EGG activity mimicking the effect of breast milk in preterm infants (Indrio *et al*., 2009).

The precise role of EGG in the clinical evaluation of patients or monitoring of therapeutic response to medications, and how this test adds to the information obtained from a gastric emptying test, remain the subject of ongoing research.

The pig, as a representative of the omnivore, is relatively close to man in a number of metabolic and physiological indicators (Květina *et al*., 1999; Nobilis *et al*., 2003; Anzenbacherová *et al*., 2003). It is not uncommon that prediction focusing on the transfer of knowledge towards human drug therapy is based on precisely this experimental species (Květina *et al*., 2009). Regarding body mass index, in the case of use of small adult pigs in a body weight range of 30–40 kg, the proportions of passive (fat) and active (muscle) masses are comparable to an adult man. Conversion of doses of studied xenobiotics and even manner and form of their administration becomes relatively comparable (Květina *et al*., 2008b). Contrary to clinical observations, experimental design allows for standardisation of the experiment set and identification and definition of the test conditions.

## Electrogastrography in experimental pigs

The small adult pig can be used in various preclinical experiments as a representative of the omnivore due to its relatively very similar gastrointestinal functions in comparison to man (Kararli, 1995). However, there are some distinct differences in the anatomy and physiology of the stomach between humans and pigs (Bureš *et al*., 2009; Kopáčová *et al*., 2010; Květina *et al*., 2008a). The porcine stomach is pouch-shaped and the gastric cardia is close to the pylorus. A special transverse pyloric fold (torus pyloricus) serves as a “gate-keeper” (Kopáčová *et al*., 2010). Gastric emptying of pigs is much slower, put through small separated amounts. There are significant remnants of food in the porcine stomach even after 36–48hours of fasting (Kopáčová *et al*., 2010; Tachecí *et al*., 2009).

To the best of our knowledge, there are no previous reports on EGG in pigs in the available literature. Our group recently set up and worked out the methods for EGG in experimental pigs (Varayil *et al*., 2009). All EGG recordings were carried out under general anaesthesia (introduction: intramuscular administration of ketamin and azaperone, repeated doses of thiopental when appropriate).

**Figure 1:**
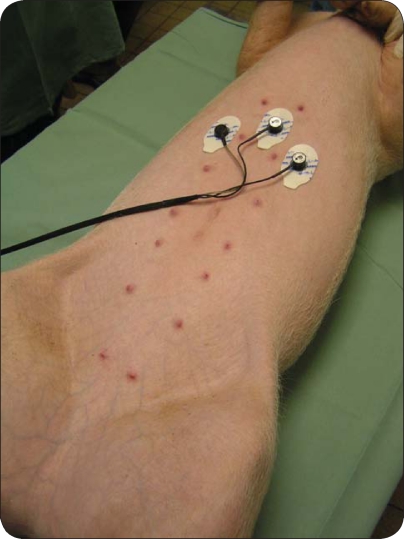
Electrogastrography in experimental pigs. General arrangement of the electrodes placement for EGG recording.

**Figure 2:**
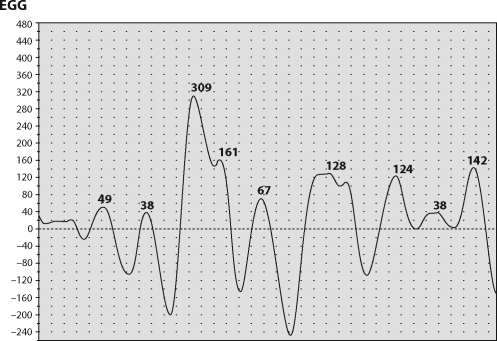
EGG rhythm of three cycles per minute at online recording.

**Figure 3:**
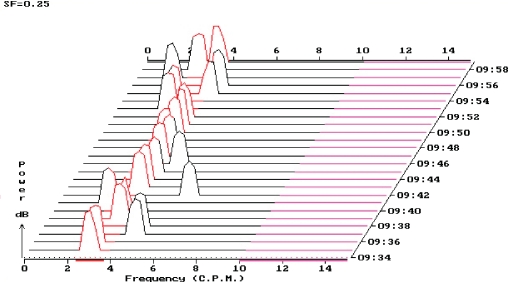
Protocol of EGG recording with prevailing rhythm of three cycles per minute (60% running spectrum percent activity).

**Figure 4:**
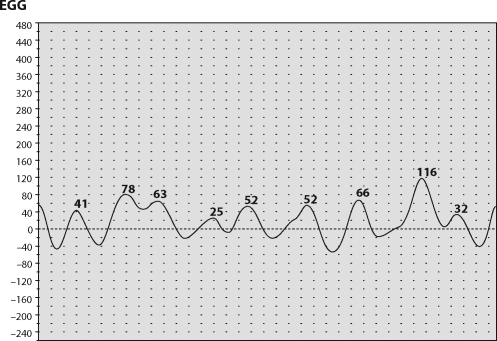
Pattern of bradygastria at online EGG recording.

**Figure 5:**
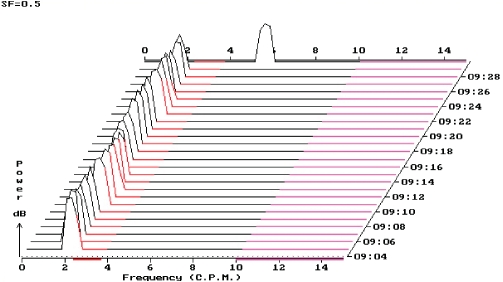
Protocol of EGG recording with prevailing bradygastria (96% running spectrum percent activity).

**Figure 6:**
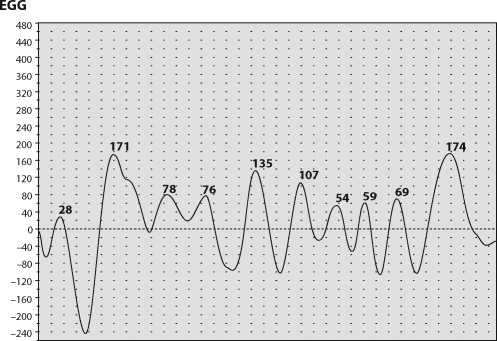
Pattern of tachygastria at online EGG recording.

**Figure 7:**
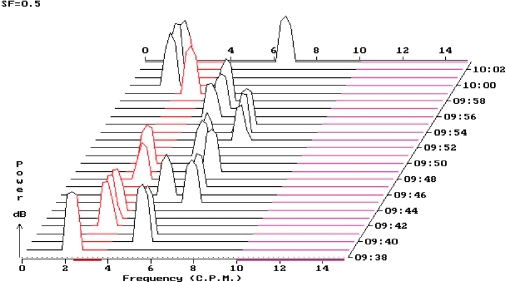
Protocol of EGG recording with prevailing tachygastria (60% running spectrum percent activity).

In our setting, all animals were lying in a right lateral position during the EGG recording. The epigastric area was shaved before application of electrodes to decrease impedance in signal conduction through the skin. Electrode placement always began with placing the first electrode roughly within 5cm of the xyphoid process in the centre and then subsequently placing the other 2 roughly at a distance of 15cm from the central electrode in left and right hypochondrium respectively (Figure 1). After connecting the device the recording was started and the animals were closely monitored for any flinging movement. A single EGG recording always lasted 30 minutes. All possible artefacts (especially motion ones) must be removed before the final evaluation. We used a running spectral analysis (based on Fourier transform) for the evaluation of the experimental EGG. Results are expressed as running spectrum percent activity. All low, medium and high frequencies of gastric slow waves were found in particular animals (Figures 2–7). The normal dominant frequency that we found in pigs in this project (3.3 ± 0.5 cycles per minute) is fully comparable with those in adult humans (Varayil *et al*., 2009). Thus our original assumption was proven – the young adult pig is a suitable model for experimental EGG.

## Volume challenge

The water load test is a standardised test to induce gastric distension and to evoke gastric motility responses without the complex hormonal response of a caloric test meal. EGG with water load test has been validated as being reliable and reproducible in humans (Koch *et al*., 2000; Chen *et al*., 2006).

In our recent EGG study in experimental pigs, we decided on volume challenge of 360mL water that is comparable with 500mL usually used in adult humans. The mean dominant frequency after volume challenge was significantly higher compared with the basic measurement (Varayil *et al*., 2009). Several studies performed previously in humans have shown that volume overload after drinking water generally affects both dominant frequency (to tachygastria) and dominant power (characterised by an increase in amplitude) which has been attributed to factors such as 1) gastric distension 2) gastric displacement 3) slow wave changes and/or 4) neurohumoral mechanisms (Chen *et al*., 2006; Friesen *et al*., 2007; Jones *et al*., 2003; Lin *et al*., 2000).

## Itopride

Itopride is a dopamine D2 antagonist with acetylcholinesterase inhibitory activity that has prokinetic effects and probably effects on gastric accommodation and hypersensitivity (Longstreth, 2010).

We used itopride as a model prokinetic drug in experimental pigs. EGG recording was carried out immediately after intragastric administration of 100 mg itopride (*i.e.* ~3mg/kg; corresponding to the maximum single dose for man). There was no obvious change in dominant frequency during subsequent EGG recording (Varayil *et al*., 2009). However, it is possible that the dose was not big enough and/or there was not the required time to make it possible for itopride to exert its prokinetic effect. Itopride has linear kinetics. In humans, maximum plasmatic concentration (t-max) of itopride is reached at about 45 minutes after oral administration (half-time of elimination is 6 hours). Gastric emptying and start of intestinal absorption of itopride might be delayed under general anaesthesia (Schurizek, 1991; Umenai *et al*., 2009). Iwanaga *et al*. (1996) studied the gastroprokinetic effect of itopride in conscious dogs. At a dose of 3 mg/kg, itopride did not affect gastrointestinal motility. With itopride 10 mg/kg the contractile force of the gastric antrum was increased (doubled) within 5 minutes after intra-duodenal administration of itopride (Iwanaga *et al*., 1996).

## Erythromycin

Macrolides are a group of closely related antibiotics characterised by a 14-, 15- or 16-membered lactone ring. Macrolides have been found to have pharmacodynamic properties beyond their antimicrobial mode of action, like anti-inflammatory, immunomodulatory and gastrointestinal prokinetic effects (Hawkyard & Koerner, 2007). Erythromycin is the most potent prokinetic drug available nowadays. Erythromycin has been shown to initiate gastric interdigestive migrating motor complexes, which are the motor events responsible for gastric emptying of indigestible solids. Erythromycin induces high amplitude gastric propulsive contractions that literally dump solid residue, including non-digestible materials, out of the stomach (Prather *et al*., 1993; Keshavarzian, 1993; Curry *et al*., 2001). Erythromycin also stimulates fundic contractility, or at least inhibits the accommodation response of the proximal stomach after food ingestion (Bruley des Varannes *et al*., 1995; Fraser & Mittal, 1994; Curry *et al*., 2001; Camilleri, 2010). Erythromycin seems to have a different mechanism of action in the stomach compared to the duodenum (Mathis & Malbert, 1995).

Erythromycin is used especially in the treatment of diabetic gastroparesis in humans (Camilleri, 2010). A systematic review identified 35 clinical trials involving erythromycin for gastroparesis of which five fulfilled inclusion criteria for the review (Maganti, 2003). All studies were small (≤13 subjects), of short duration (≤4 weeks) and had methodological weaknesses. Nevertheless, improvement was reported in 26 of 60 patients (43%) (Maganti *et al*., 2003). Van der Voort *et al*. (2003) investigated functional dyspepsia, irritable bowel syndrome and healthy controls. Disturbed gastric emptying correlated with a lack of postprandial increase in the EGG amplitude. Prokinetic erythromycin improved both gastric emptying and gastric electrical activity (van der Voort *et al*., 2003).

In our latest project, we used EGG to test the effect of erythromycin on gastric myoelectrical activity in experimental pigs. Intragastric administration of a therapeutic dose of erythromycin (1600 mg) substantially increased the gastric myoelectrical activity. There was a significant prokinetic effect of erythromycin (compared to baseline recording): a statistically significant increase in dominant frequency at recordings starting 90 minutes with maximum 330 minutes after erythromycin administration. In a human study in healthy volunteers, low dose i.v. erythromycin also achieved an increase in percent tachygastria (DiBaise *et al*., 2001).

## Tasks and perspectives for future

Further improvements in methods of EGG in experimental pigs are needed. Optimal placement of leads must be searched for before each recording (different for drug testing and volume challenge). Power analysis must become an inherent part of each evaluation. It will be also useful to verify and validate if the ratio of antral motor index is beneficial in experimental pigs. This index is calculated as the number of waves the sum of amplitudes (Faure *et al*., 2000). And last but not least, standard protocol of EGG in experimental pigs must be completed.

Not only several drugs, but also different probiotic bacteria are to be tested by means of EGG in the near future. Experimental EGG is an optimal non-invasive method to investigate the motor effect of particular drugs.

## Conclusions

EGG in experimental pigs is feasible. The mean dominant frequency in pigs is comparable with that found in humans. Experimental EGG is an important basis for further preclinical projects in pharmacology and toxicology.
